# Supplementary material for: Role of p110a subunit of PI3-kinase in skeletal muscle mitochondrial homeostasis and metabolism
Source: Nat Commun. 2019 Jul 30;10:3412. doi: 10.1038/s41467-019-11265-y (PMC6667496; doi:10.1038/s41467-019-11265-y)
Supplement: Supplementary file 1 — Supplementary Information [file 41467_2019_11265_MOESM1_ESM.pdf]

Supplementary Information

Title: Role of p110a subunit of PI3-kinase in skeletal muscle mitochondria

Authors: Li et al .

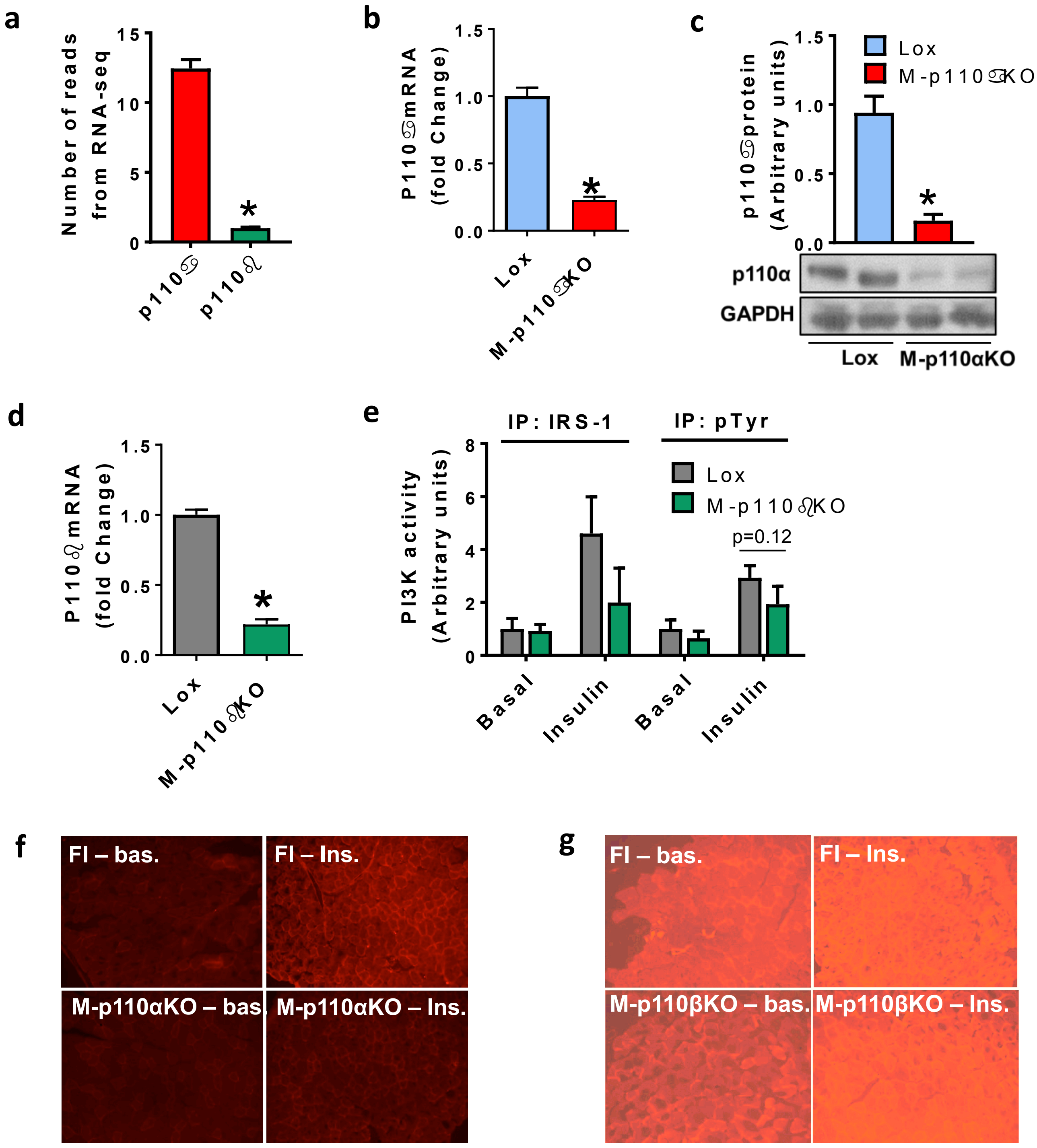

Supplementary Figure 1. p110α recombination blunts PI3K activity in M-p110αKO

a Number of reads of p110α and p110β genes from TA muscle of p110α-floxed and p110β floxed mice measured by RNA-Seq analysis (n = 4).

b mRNA expression of p110α gene in pooled hindlimb skeletal muscles of M-p110αKO and control mice (n = 4).

c Representative western blot and densitometric analysis of p110α protein content in TA muscle of M-p110αKO and control mice (n = 6).

d mRNA expression of p110β gene in major hindlimb skeletal muscles of M-p110βKO and control mice (n = 5).

e PI3K activities from extracts that were immunoprecipitated with anti-IRS-1 or anti-pTyr in gastrocnemius muscles of M-p110βKO and control mice after insulin injection (5 U of regular insulin via inferior vena cava) (n = 4).

f and **g** Phosphatidylinositol (3,4,5)-trisphosphate (PIP3) immunostaining of TA muscles from M-p110αKO (f) and M-p110βKO (g) mice and their controls after insulin injection (5 U of regular insulin via inferior vena cava) (n = 4).

All mice were 3-month-old. PIP3, Phosphatidylinositol (3,4,5)-trisphosphate. Data are mean ±SEM.

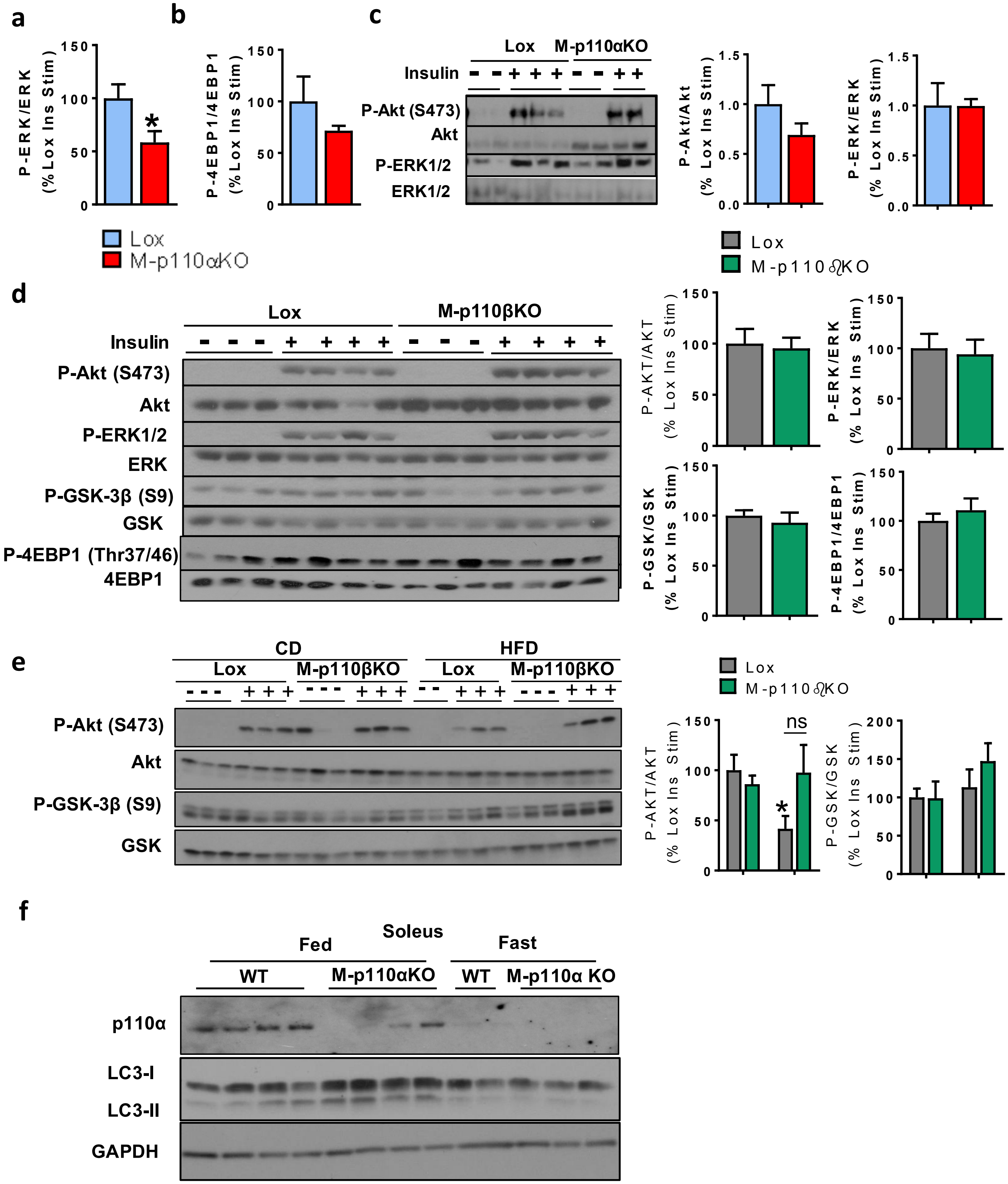
a
P-ERK/ERK (% Lox Ins Stim)
Lox
M-p110αKO
b
P-4EBP1/4EBP1 (% Lox Ins Stim)
c
Lox
M-p110αKO
Insulin
P-Akt (S473)
Akt
P-ERK1/2
ERK1/2
P-Akt/Akt (% Lox Ins Stim)
P-ERK/ERK (% Lox Ins Stim)
Lox
d
Lox
M-p110βKO
Insulin
P-Akt (S473)
Akt
P-ERK1/2
ERK
P-GSK-3β (S9)
GSK
P-4EBP1 (Thr37/46)
4EBP1
P-AKT/AKT (% Lox Ins Stim)
P-ERK/ERK (% Lox Ins Stim)
P-GSK/GSK (% Lox Ins Stim)
P-4EBP1/4EBP1 (% Lox Ins Stim)
e
CD
HFD
Lox
M-p110βKO
P-Akt (S473)
Akt
P-GSK-3β (S9)
GSK
Lox
ns
P-AKT/AKT (% Lox Ins Stim)
P-GSK/GSK (% Lox Ins Stim)
f
Soleus
Fed
Fast
WT
M-p110αKO
M-p110α KO
p110α
LC3-I
LC3-II
GAPDH

Supplementary Figure 2. Muscle insulin signaling of M-p110αKO and M-p110βKO

a and **b** Densitometric analysis of insulin signaling assessed by western blot (Fig. 1b) in quadriceps muscle of M-p110αKO and control mice after insulin injection.

c Western blot and densitometric analysis of insulin signaling in quadriceps muscles of M-p110αKO and control mice on a 60% of HFD for 13 weeks.

d and **e** Western blot and densitometric analysis of insulin signaling in quadriceps muscles of M-p110βKO and control mice on a CD (d) or 60% of HFD (e) for 15 weeks.

f Western blot and densitometric analysis of FoxO proteins in TA muscles of 3-month-old M-p110αKO and control mice.

All mice were 3-to 4-month-old. Mice from Fig. S2a to S2e were fasted overnight and treated with saline or insulin intravenously as described in the EXPERIMENTAL PROCEDURES. * $P < 0.05$ by Student's t-test. Light blue bars represent p110α-floxed mice; red bars represent M-p110αKO mice; grey bars represent p110β-floxed mice; Green represent M-p110βKO mice. Data are mean ±SEM.

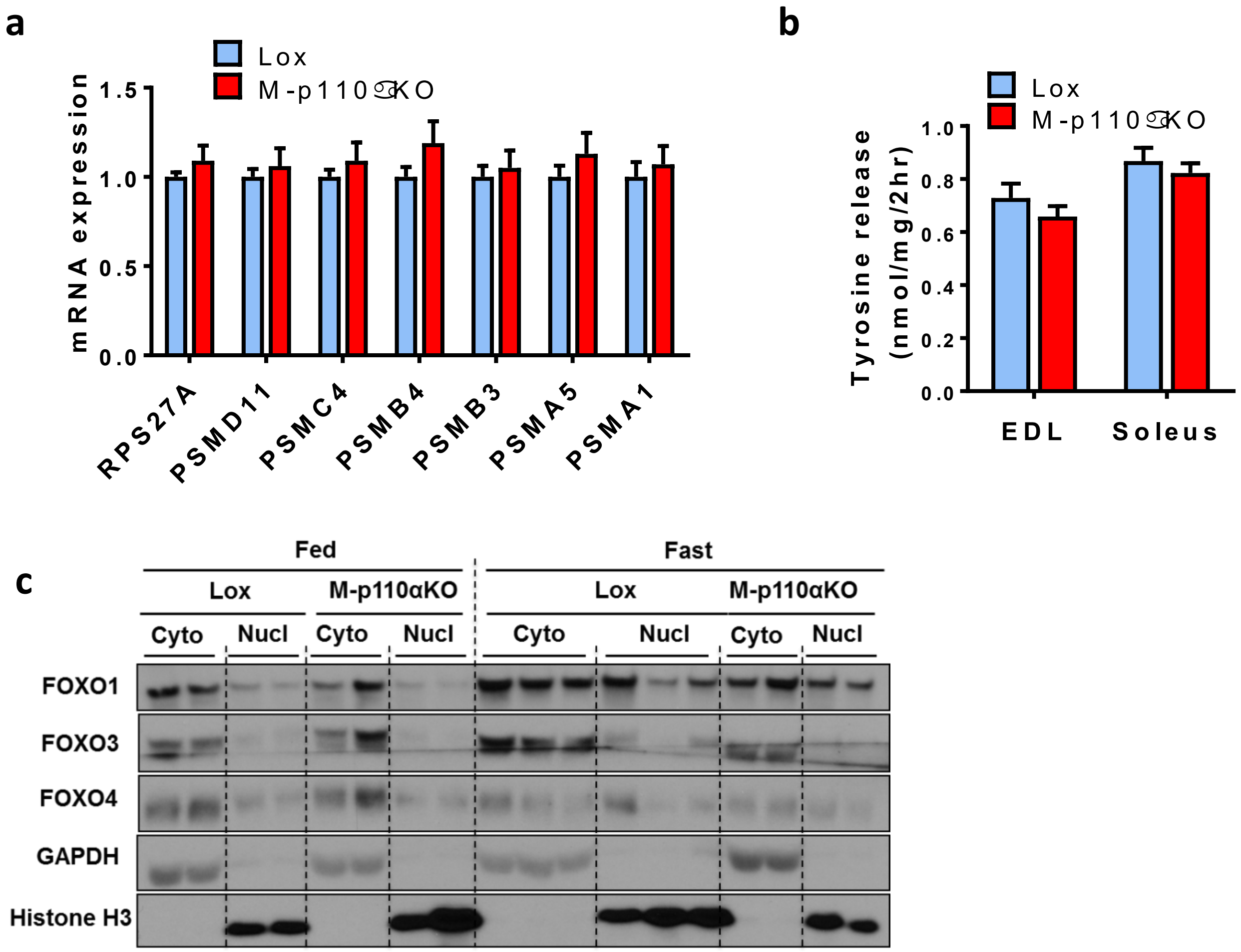

Supplementary Figure 3. Unchanged mRNA expression of genes that encode proteasomal subunit proteins and proteolysis in young M-p110αKO mice.

a mRNA expression of genes that codes for proteasomal subunits, isolated from TA muscles of 3-month-old M-p110αKO and control mice (n = 4).

b Proteolysis rate measured by tyrosine release *ex vivo* in EDL and soleus muscles isolated from fed control and M-p110αKO mice at 5 months of age (n = 8-9).

Data are mean ±SEM.

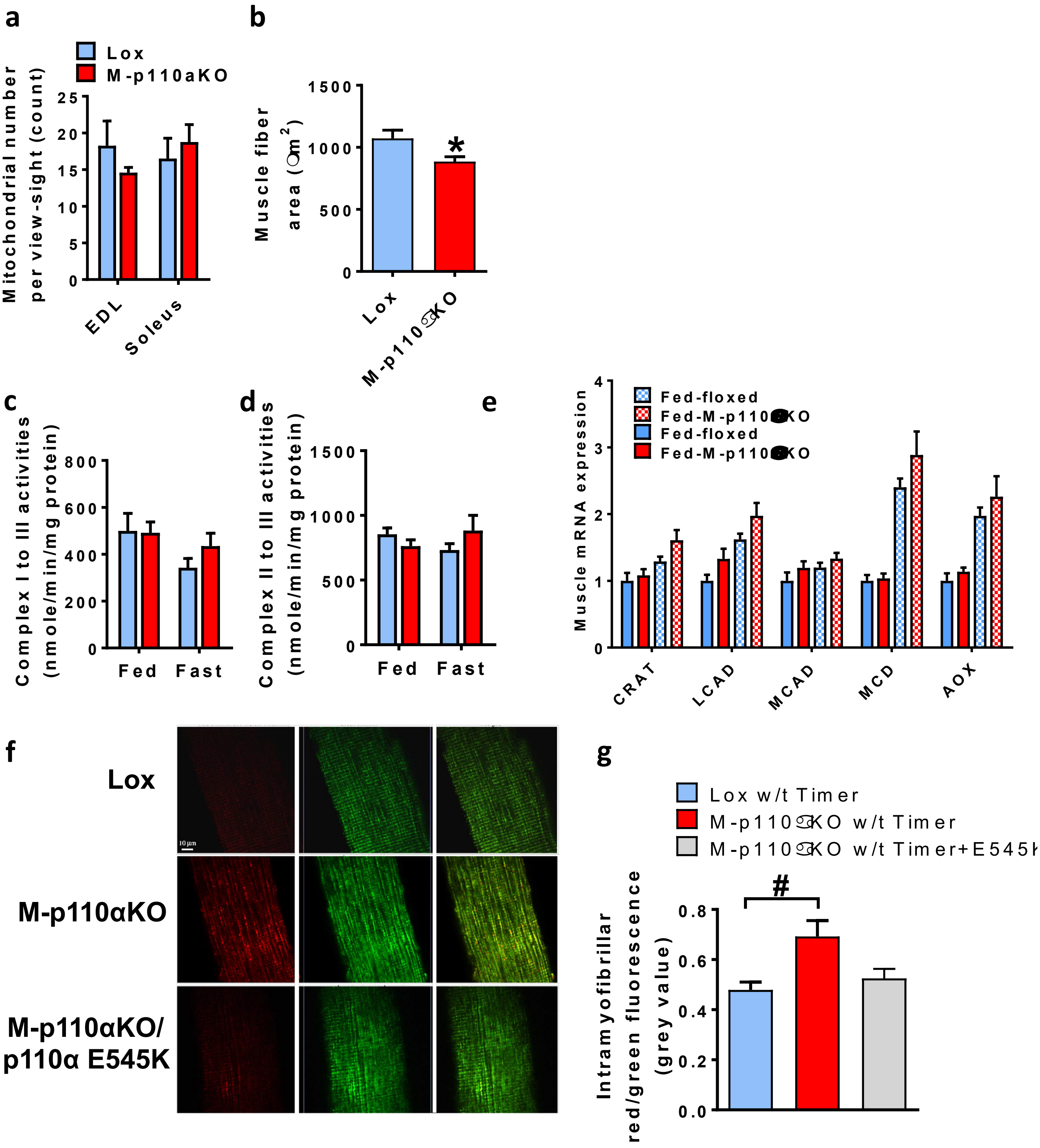

Supplementary Figure 4. Mitochondrial number and Complex I-III activities are unchanged, but Mito-Timer studies indicate increased Oxidant level in M-P110αKO muscle

a Mitochondrial numbers per view-sight were quantified in muscles of M-p110αKO and control mice from electron microscope (EM) in Figure. 3a (n = 4).

b mito-GFP was transfected into quadriceps muscle of M-p110αKO and control mice and visualized *in vivo* 5 days later as described in EXPERIMENTAL PROCEDURES. Muscle fiber areas per view-sight were quantified from intramyofibrillar positions in muscles of M-p110αKO and control mice (n = 5-6).

c and **d** Mitochondrial respiratory enzyme Complex I to III (c) and II to III activities (d) in mitochondria isolated from skeletal muscle of M-p110αKO and control mice in fed or 24hr fasted conditions (n =6-7).

e mRNA expression of genes regulating and β-oxidation in TA muscles of M-p110αKO and control mice fasted for 24 hrs or randomly fed (n = 8).

f pMitoTimer was *in situ* transfected into quadriceps muscle fibers of living mice using a gene gun delivery approach, and visualized *in vivo* 5 days later by confocal microscope (upper and middle set of images). A different cohort of M-p110αKO mice was co-transfected with constitutively active mutant p110α (PIK3CA) E545K into quadriceps of these mice (bottom set of images).

g Red/green fluorescence in intramyofibrillar position of quadriceps muscles in (c) (n = 4 with 3-4 fibers per mouse).

All mice were 4- to 5-month-old. # $P < 0.05$ by one-way ANOVA. Light blue bars represent p110α-floxed mice; red bars represent M-p110αKO mice. Intromyo., intromyofibriallar. Timer, pMitoTimer; E545K, p110α (PIK3CA) E545K; Data are mean ±SEM.

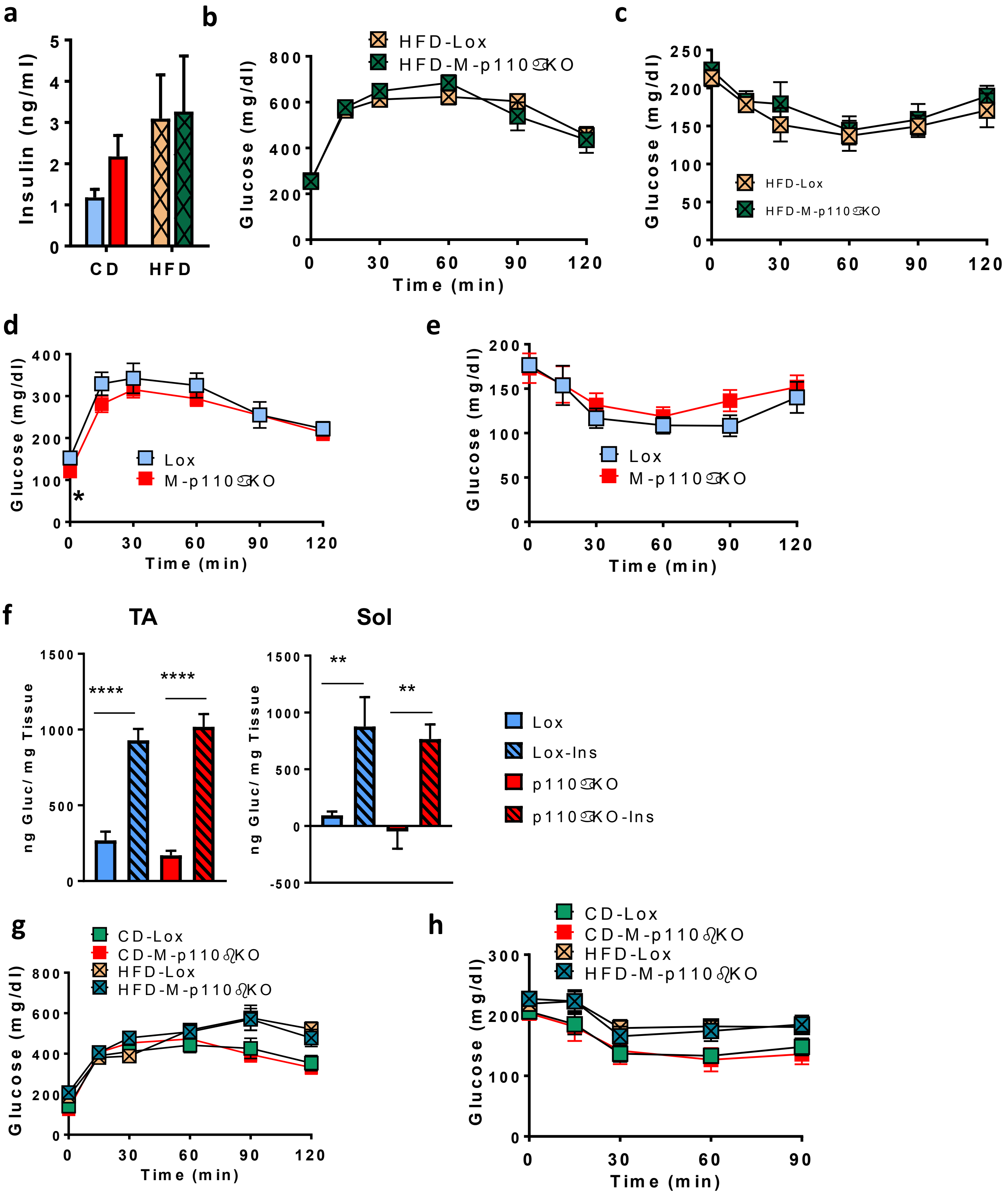
a
Insulin (ng/ml)
CD
HFD
b
HFD-Lox
HFD-M-p110KO
Glucose (mg/dl)
Time (min)
c
Glucose (mg/dl)
HFD-Lox
HFD-M-p110KO
d
Glucose (mg/dl)
Lox
M-p110KO
*
Time (min)
e
Glucose (mg/dl)
Lox
M-p110KO
Time (min)
f
TA
Sol
ng Gluc/ mg Tissue
****
**
Lox
Lox-Ins
p110KO
p110KO-Ins
g
CD-Lox
CD-M-p110KO
HFD-Lox
HFD-M-p110KO
Glucose (mg/dl)
Time (min)
h
CD-Lox
CD-M-p110KO
HFD-Lox
HFD-M-p110KO
Glucose (mg/dl)
Time (min)

Supplementary Figure 5. Whole body glucose and insulin tolerance are not impaired in M-p110αKO or M-p110βKO mice on chow diet or 60% of High-Fat Diet.

a Serum insulin levels from 3-month-old M-p110αKO and control mice under CD or 60% of HFD for 15 weeks (n = 4-8).

b Intraperitoneal glucose tolerance test (GTT) performed in 3-month-old M-p110αKO and control mice on 60% of HFD for 15 weeks (n = 5-8).

c Intraperitoneal insulin tolerance (ITT) were performed in 3-month-old M-p110αKO and control mice on CD or 60% of HFD for 15 weeks (n = 5-8).

d and **e** Intraperitoneal GTT (D) and ITT (E) were performed in M-p110αKO and control mice at 16 months of age (n = 8).

f and **g** Intraperitoneal GTT (f) and ITT (g) were performed in 3-month-old M-p110βKO and control mice on chow diet (CD) or 60% of High-fat diet (HFD) for 15 weeks (n = 6-8).

* $P < 0.05$ by Student's t-test. Light blue bars represent p110α-floxed mice; red bars represent M-p110αKO mice. Data are mean ±SEM.

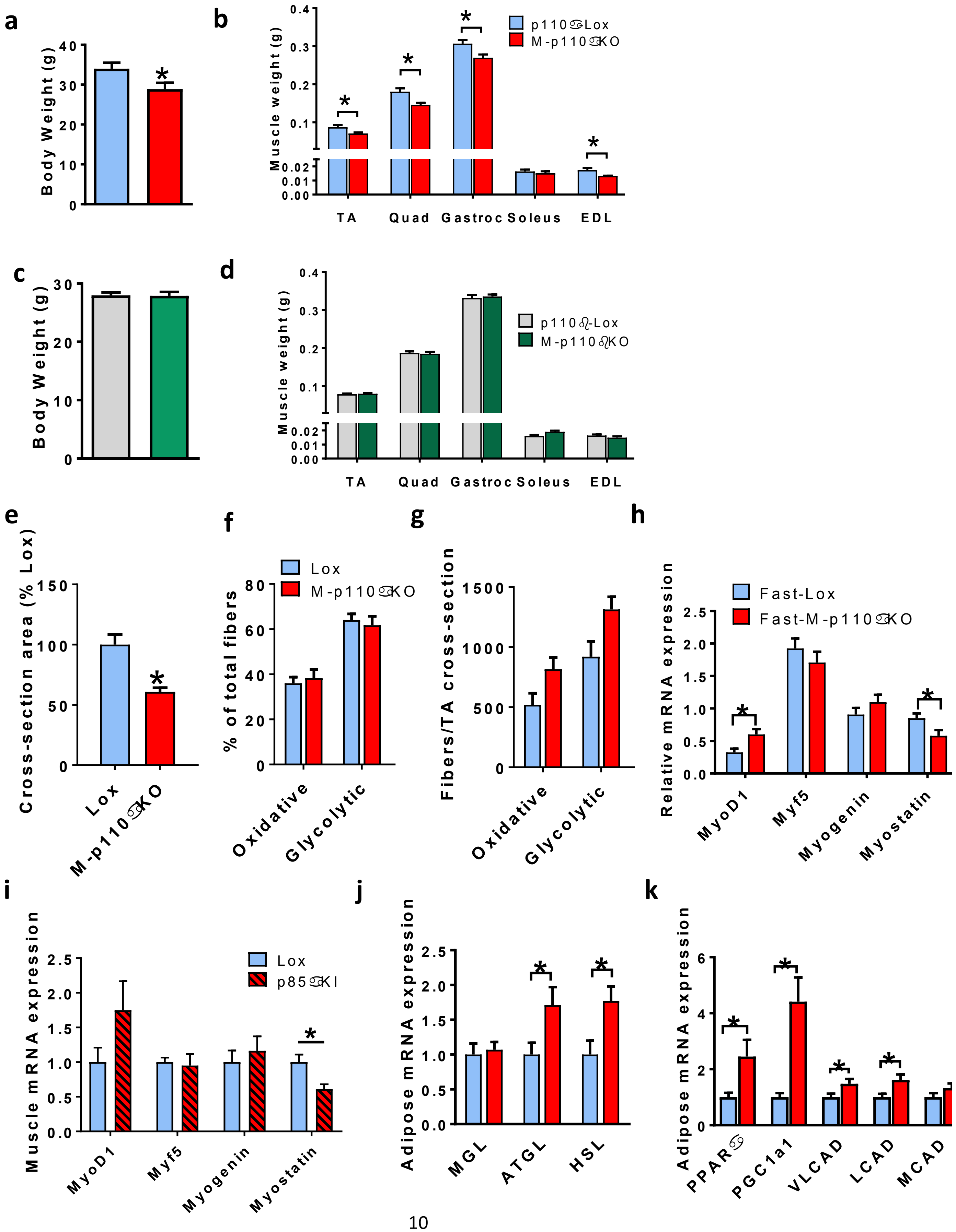
a
Body Weight (g)
b
Muscle weight (g)
TA
Quad
Gastroc
Soleus
EDL
c
Body Weight (g)
d
Muscle weight (g)
e
Cross-section area (% Lox)
Lox
f
% of total fibers
Lox
Oxidative
Glycolytic
g
Fibers/TA cross-section
Oxidative
Glycolytic
h
Relative mRNA expression
Fast-Lox
MyoD1
Myf5
Myogenin
Myostatin
i
Muscle mRNA expression
Lox
MyoD1
Myf5
Myogenin
Myostatin
j
Adipose mRNA expression
MGL
ATGL
HSL
k
Adipose mRNA expression
PGC1a1
VLCAD
LCAD
MCAD

Supplementary Figure 6. Decreased muscle mass and increased mRNA expression of genes regulating lipolysis and fatty acid oxidation in adipose tissue of M-p110αKO Mice.

a Body weights from 3-month-old M-p110αKO and control mice (n = 8).

b Muscle weights from 24-month-old M-p110αKO and control mice (n = 5-6).

c Body weights from 3-month-old M-p110βKO and control mice (n = 8).

d Muscle fiber cross-section area of TA muscles from 3-month-old M-p110αKO and control mice from SDH stain in Figure 6B. (n = 5).

e and **f** Percentage (e) and total number (f) of oxidative and glycolytic fibers in TA muscle cross-section of M-p110αKO and control mice from SDH stain in Figure 6B (n = 5).

g mRNA expression of myogenic factors in TA muscles of 3-month-old M-p110αKO and control mice fasted for 24 hrs (n = 5).

h and **i** mRNA expression of genes regulating lipolysis (h) and β-oxidation (i) in epididymal fat of M-p110αKO and control mice after 24 hrs fasting (n = 8).

* $P < 0.05$ by Student's t-test. Light blue bars represent p110α-floxed mice; red bars represent M-p110αKO mice. TA, tibialis anterior; Gastroc, gastrocnemius; Quad, quadriceps; EDL, extensor digitorum longus. Data are mean ±SEM.

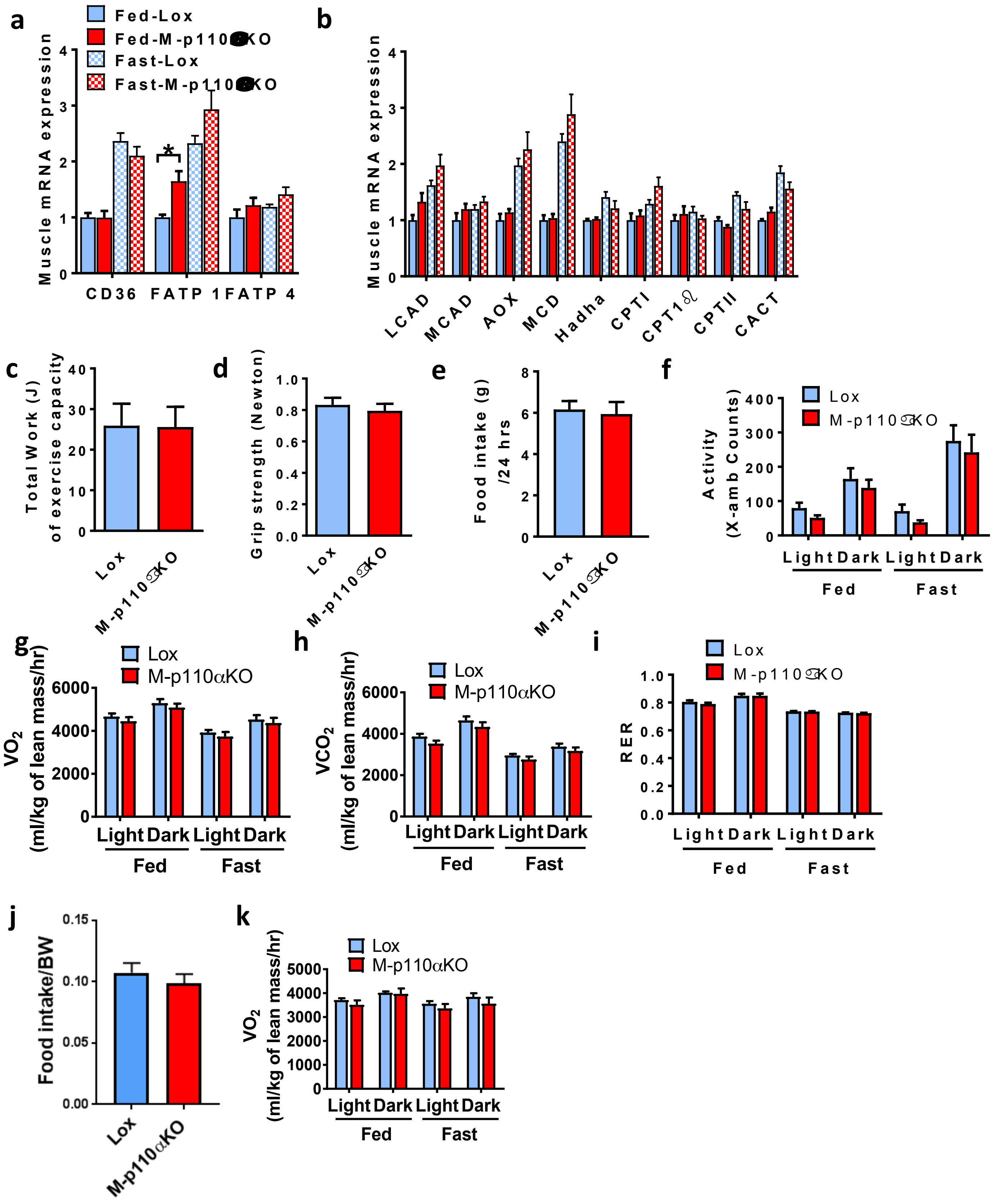
a
Fed-Lox
Fast-Lox
Muscle mRNA expression
CD36
FATP 1
FATP 4
b
Muscle mRNA expression
LCAD
MCAD
AOX
MCD
Hadha
CPTI
CPTII
CACT
c
Total Work (J) of exercise capacity
Lox
d
Grip strength (Newton)
Lox
e
Food intake (g) /24 hrs
Lox
f
Lox
Activity (X-amb Counts)
Light Dark Light Dark
Fed
Fast
g
Lox
M-p110αKO
VO2 (ml/kg of lean mass/hr)
Light Dark Light Dark
Fed
Fast
h
Lox
M-p110αKO
VCO2 (ml/kg of lean mass/hr)
Light Dark Light Dark
Fed
Fast
i
Lox
RER
Light Dark Light Dark
Fed
Fast
j
Food intake/BW
Lox
M-p110αKO
k
Lox
M-p110αKO
VO2 (ml/kg of lean mass/hr)
Light Dark Light Dark
Fed
Fast

Supplementary Figure 7. Metabolic parameters of 3- and 24-month-old M-p110αKO mice assessed by CLAMS metabolic Cages.

a mRNA expression of fatty acid transports in TA muscles of M-p110αKO and control mice fasted for 24 hrs or randomly fed (n = 4-9).

b mRNA expression of genes regulating lipolysis in TA muscles of M-p110αKO and control mice fasted for 24 hrs or randomly fed (n = 8).

c Total work of 3-month-old M-p110αKO and control mice from acute treadmill test (n = 6).

d Forelimb grip strength of M-p110αKO and control mice at 3 months of age (n = 6).

e Accumulative food intake in 3-month-old M-p110αKO and control mice over 24 hr in CLAMS metabolic cages (n = 8).

f-i Spontaneous activities (f), VO_2 (g), VCO_2 (h) and respiratory exchange ratio (RER) (i) from 3-month-old M-p110αKO and control mice during fed and fast cycles over 48hr in CLAMS metabolic cages (n = 8).

j Accumulative food intake in 24-month-old M-p110αKO and control mice over 24hr in CLAMS metabolic cages (n = 5-6).

k VO_2 from 24-month-old M-p110αKO and control mice during fed and fast cycles over 48hr in CLAMS metabolic cages (n = 5-6).

* $P < 0.05$ by Student's t-test. Data are mean ±SEM.

Fig. 1b

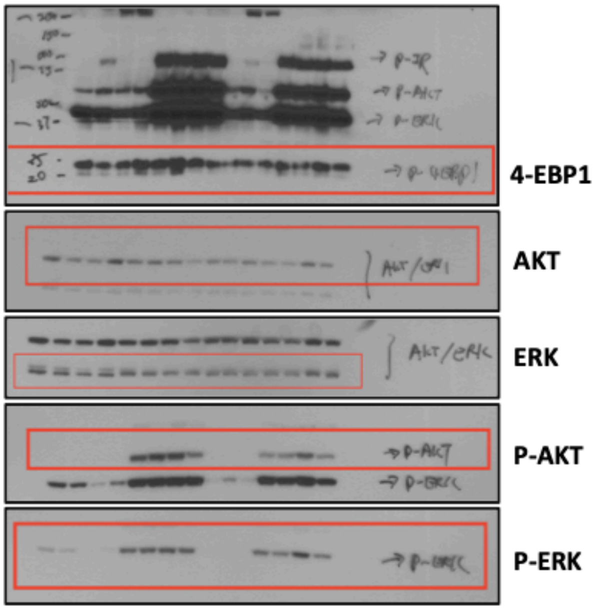

Fig. 2a

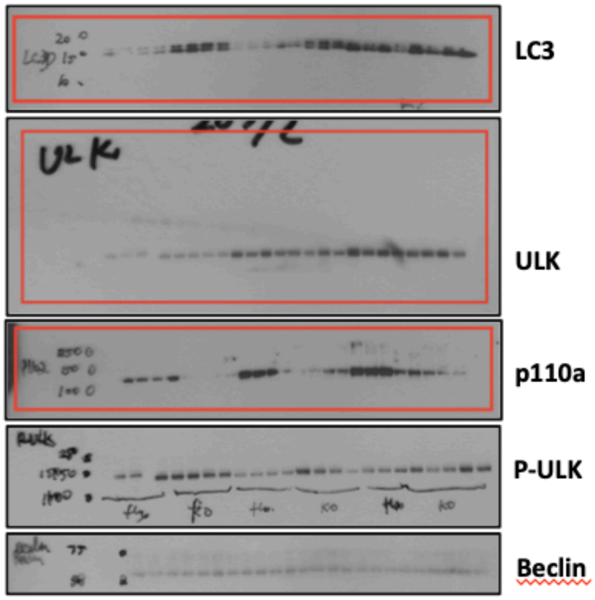

Fig. 3e

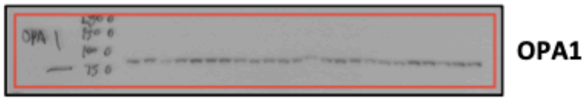

Fig. 4d

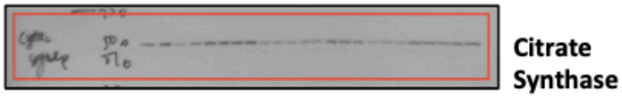

Fig. 4a

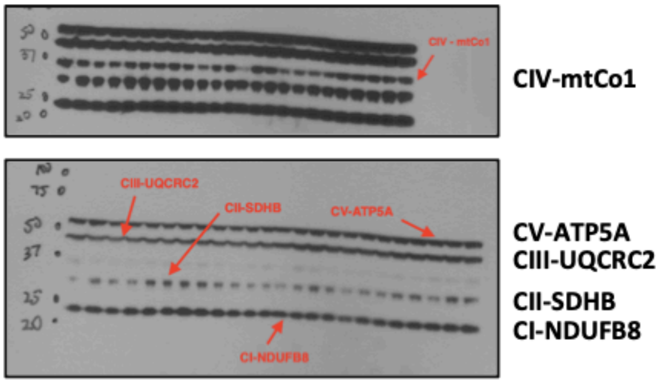

Supplementary Figure 8. Uncropped blots.

Supplementary Table 1 Primers for quantitative RT-PCR of mouse genes

Common name	Gene name	5' primer	3' primer
AOX	*Acox1*	CCTGATTCAGCAAGGTAGGG	TCGCAGACCCTGAAGAAATC
ATGL	*Pnpla2*	TAATGTTGGCACCTGCTTCA	CCACTCACATCTACGGAGCC
Beclin1	*Becn1*	GGCGAGTTTCAATAAATGGC	CCAGGAACTCACAGCTCCAT
Cact	*Slc25a20*	AAACTTGGTGGTTGTGTCTGC	TTTAAGAACCTCCTGGCTGG
Cathepsin L	*Ctsl*	TATCCCTCAGCAAGAGAAAGCCCT	TCCTTCATAGCCATAGCCCACCAA
CD36	*Cd36*	CCTGCAAATGTCAGAGGAAA	GCGACATGATTAATGGCACA
CPT1β	*Cpt1b*	GCTGCTTGCACATTTGTGTT	TGTCTACCTCCGAAGCAGGA
CPTI	*Cpt1a*	AGTGGCCTCACAGACTCCAG	GCCCATGTTGTACAGCTTCC
CPTII	*Cpt2*	TGGCTGTCATTCAAGAGAGG	ATGCACTACCAGGACAGCCT
FAS	*Fas*	CCTCAGCTTTAAACTCTCGGA	CAGACATGCTGTGGATCTGG
FATP1	*Slc27a1*	AGCCGAACACGAATCAGAAC	TTCTGTGTGTACGTGGGTGG
FATP4	*Slc27a4*	TCCTTCCGCAACTCTGTCTT	AAGGAGCTGCCTCTGTATGC
Gabarapl 1	*Gabarapl 1*	GTCATCGTGGAGAAGGCTCCTAAA	GGAGGGATGGTGTTGTTGACAAAG
GAPDH	*Gapdh*	TGTCGTGGAGTCTACTGGTGTCTT	TCTCGTGGTTCACACCCATCACAA
Hadha	*Hadha*	CTGGTCAGCAGAGCAGAAGA	ATTGGCAGTCTCAGTCGCTT
HSL	*Lipe*	GGAGAGAGTCTGCAGGAACG	CCTGCAAGAGTATGTCACGC
Lamp2a	*Lamp2*	ACAACCTGACTCCTGTCGTTCAGA	AGTTGGAGTTGGAGTGGGTGTTGA
LC3B	*Map1lc3b*	CACTGCTCTGTCTTGTGTAGGTTG	TCGTTGTGCCTTTATTAGTGCATC
LCAD	*Acadl*	TCTTGCGATCAGCTCTTTCA	GGTACATGTGGGAGTACCCG
MCAD	*Acadm*	GCGAGCAGAAATGAAACTCC	AGCTCTAGACGAAGCCACGA
MCAD	*Acadm*	GCGAGCAGAAATGAAACTCC	AGCTCTAGACGAAGCCACGA
MCD	*Mlycd*	GCTACCAGGCTGAGGATCTG	CCTCATGGTCAACTACCGCT
MGL	*Mgll*	CACTTTTCCAGAACACACCC	TGACTTTGCTCGGGGACC
Myf5	*Myf5*	AAT GCC ATC CGC TAC ATT GAG AGC	TGT CAA AGC TGC TGT TCT TTC GGG
Myod1	*Myod1*	AGC ACT ACA GTG GCG ACT CAG AT	TCC ACT ATG CTG GAC AGG CAG T
Myogenin	*Myog*	TTG CTC AGC TCC CTC AAC CAG GA	AGA TTG TGG GCG TCT GTA GGG TCA
Myostatin	*Mstn*	TGG CTC AAA CAG CCT GAA TCC AAC	TGG GTG TGT CTG TCA CCT TGA CTT
p110α	*PIK3CA*	ACTTTGTGACCTTCGGCTT	TCTTGGACTTCTGGATCTTTAACC
p110β	*PIK3CB*	GATGTCAAGGTTGTCTGCCA	AGAAGACTGAAATGGGGGCT
PGC1α1	*Ppargc1a1*	CACTTCAATCCACCCAGAAAGCT	GGACATGTGCAGCCAAGACTCT
PGC1α2	*Ppargc1a2*	GTTCAGGAAGATCTGGGCAAA	CCACCAGAATGAGTGACATGGA
PGC1α3	*Ppargc1a3*	TTCAGGAAGATCTGGGCAAAGA	AAGTGAGTAACCGGAGGCATTC
PGC1α4	*Ppargc1a4*	TCACACCAAACCCACAGAAA	CTGGAAGATATGGCACAT
PPARα	*Ppara*	CAGTGGGGAGAGAGGACAGA	AGTTCGGGAACAAGACGTTG

PSMA1	*Psma1*	GACGAGACACAGGAAGTGGTC	CAATTGCGGGTCTAACTGCT
PSMA5	*Psma5*	TGTCCTTGATCACCTCCTCC	GAAGCTGAATGCAACCAACA
PSMB3	*Psmb3*	GAGCAAATGAAGGGCTTGAA	GGCCAACCTCCTGTATGAGA
PSMB4	*Psmb4*	TGTGGACATGCTTGGTGTAG	CTGGCTGCTTCTCTAGAACTT
PSMC4	*Psmc4*	CTTGGAAGCTGTGGATCAGAA	TCCCGGTCGATGGTACTC
PSMD11	*Psmd11*	CTAGATATGGAAGCAGCCACAG	CCAATGCTTGGCGTAAGAAAG
RPS27A	*Rps27a*	TCCTGGATCTTGGCCTTTAC	CCACGATGCAGATCTTTGTG
VLCAD	*Acadvl*	CTGATGAGCTCCCAGGGTAA	TTGGGCCTCTCTAATACCCA

SUPPLEMENTAL EXPERIMENTAL PROCEDURES

Animal Diets and Treatments

Animals were maintained on a standard chow diet containing 22% of calories from fat (Lab Diet 9F, 5020) or given a high-fat diet (HFD) with 60% calories from fat (OpenSource Diet D 12492, Research Diet). All mice were housed in a 20-22°C temperature-controlled room on a 12 h-light/dark cycle, and were allowed ad libitum access to water and food. For fasting studies, mice were transferred to a new cage without food for 24 hours and then sacrificed. Genome scan analysis carried by Jackson Laboratory showed p110α-floxed mice in our study was 97.4±0.1 % C57BL/6 mice, whereas the p110β-floxed mice were 74.7±0.8 % C57BL/6.

Phosphatidylinositol 3-kinase (PI3K) activity

Gastrocnemius muscles were collected from M-p110αKO and control mice that had been injected with 5 U of regular insulin (Novolin, Novo Nordisk) via inferior vena cava and snap frozen in liquid nitrogen. PI3K activity was measured using a PI3-Kinase Activity ELISA kit (Echelon, K-1000s) following the manufacturer's instructions. Briefly, muscle were homogenized in ice-cold PI3K assay lysis buffer, and 500 μg of protein were used for subsequent immunoprecipitation with anti-pTyr (BioLegend, 309302) or anti-IRS-1 (JD 287 (Sun et al., 1991)). PI3K assays were then performed as described.

Metabolic Measurements

Mice were housed individually and their metabolic activities were assessed using an OPTO-M3 sensor system (Comprehensive Laboratory Animal Monitoring System, CLAMS; Columbus

Instruments) in the Animal Physiology Core of the Joslin Diabetes Research Center (DRC). Food intake, spontaneous activity, heat production, volume of O_2 consumption and volume of CO_2 produced were measured over a 72h light and dark cycle (48h fed followed by 24h fast). Glucose tolerance tests (GTT) and insulin tolerance tests (ITT) were performed in the mice that were fasted overnight and injected with glucose intraperitoneally (2 g/kg body weight) (GTT) or those after 4 h fasting and received insulin intraperitoneally (1 mU/g body weight) (ITT) as previously described [1]. Circulating hormones were assessed using a mouse insulin ELISA kit (Crystal Chem, 90080), a GDF-8/Myostatin Quantikine ELISA Kit (R&D systems, DGDF80) or a Mouse/Rat FGF-21 Quantikine ELISA Kit (R&D systems, MF2100) respectively. *In vivo* insulin signaling was performed in anesthetized, overnight-fasted mice that had been injected with 5 U of regular human insulin (Novolin, Novo Nordisk) via inferior vena cava (IVC). 7-10 minutes later, tissues were harvested and snap frozen in liquid nitrogen.

Physiologic Measurements

Maximal exercise tolerance was measured in 3-and 16-month-old mice using a treadmill running protocol [2]. In brief, mice were given 30 minutes to acclimate to the treadmill (Columbus Instruments, Columbus, OH). They then exercised at 5 m/min at 0^0 incline for 5 min, and after each 5 min interval the speed was increased by 5 m/min until reaching 20 m/min with 0^0 incline. The slope was then increased every 5 min by 5 degrees, while maintaining the speed at 20 m/min until the mouse reached the exhaustion point. Maximal exercise tolerance was determined by the cumulative amount of work (kJ) that each mouse performed, calculated as body weight (kg) × vertical distance covered (m) × 9.81. Muscle strength of the forelimbs of each mouse was measured using a Grip Strength Meter (Columbus Instruments, Columbus, OH). In brief, each

mouse was held from the tip of its tail and the front paws grasped the grid. The grip was released when the mouse was pulled back gently. Hind limbs were kept free during the test. Each animal was tested 3 times with a 5 minute break between each measurement.

Histology

SDH Staining. Frozen cross-sections of TA muscles were stained for succinate dehydrogenase (SDH) by immersing slides in staining buffer containing PBS with 0.5 M disodium succinate, 20 mM $MgCl_2$, and 0.5 mg/ml of nitro blue tetrazolium for 15 minutes at 37˚C. All slides were stained at the same time for the same duration, followed by the termination of stain by immersing slides in PBS [3].

Haemotoxylin and eosin (H&E) Staining. Quadriceps muscles were fixed overnight with neutral-buffered 10% formalin at 4°C, paraffin embedded, sectioned, and stained with hematoxylin and eosin (H&E) [4].

LC3 Staining. Frozen cross-sections of quadriceps muscles were immunofluorescently stained for LC3A (Cell Signaling, CS 4599) as previously described [5].

PIP3 Staining. TA muscles were collected from M-p110αKO and control mice that were injected with 5 U of regular insulin (Novolin) via inferior vena cava and snap frozen in liquid nitrogen. Frozen cross-sections of these muscles were stained for PIP3 (Echelon Biosciences, Z-B345) using the protocol previously described [6].

Quantification of Muscle Histology

Images of muscles were quantified using Image J64 software (National Institutes of Health) as described previously [7]. The circumference of stained fibers was drawn manually to measure the

pixel cross-section area, and the number of fibers with specific stain was counted manually with <5% flaw/folding in entire cross-section. For the muscles stained with SHD, light to dark purple staining on any portion of the fibers was considered “Oxidative” while white to grey fibers were considered “Glycolytic”.

Triglyceride Assessment

Muscle and liver triglyceride were determined using Triglyceride Reagents Set (Pointe Scientific, HT732-200) as described [8]. Briefly, 100 mg gastrocnemius muscle or 50 mg liver were homogenized in 2:1 chloroform/methanol buffer. Extracts were evaporated for 1h prior to biochemical determination per manufacturer’s instructions.

Citrate Synthase Activity Assessment

Citrate synthase activity was determined using muscle lysates of M-p110α and control mice as previously described [9]. Briefly, 6 μg protein lysate isolated from gastrocnemius muscles of the mice was mixed with reaction buffer that contained 80 mM Tris-HCl, 0.1 mM 5,5-dithio-bis-2-nitrobenzoic acid (DTNB), 0.4 mM acetyl-CoA and 0.6 mmol/l oxaloacetate. Citrate synthase activity was detected by the transfer of sulfhydryl group to DTNB and calculated as micromoles per minute per gram of protein.

ATP Assay and ROS/RNS Assessment

ATP production was determined from muscle lysate of M-p110α and control mice using a luminescent assay kit (Molecular Probes, A22066) following the manufacturer’s instruction. Briefly, muscles were homogenized in ice-cold PBS, and muscle ATP was quantified by using a

luciferase-based kit in which luciferase converts luciferin to oxyluciferin in the presence of ATP from muscle samples. The emitted luminescence signal is directly proportional to ATP content. ROS/RNS production was assessed in muscle lysate using an In Vitro ROS/RNS Assay Kit (Cell Biolabs, STA-347).

Reference

1. Bruning, J.C., Michael, M.D., Winnay, J.N., Hayashi, T., Horsch, D., Accili, D., Goodyear, L.J., and Kahn, C.R. (1998). A muscle-specific insulin receptor knockout exhibits features of the metabolic syndrome of NIDDM without altering glucose tolerance. Mol Cell *2*, 559-569.
2. Li, M., Vienberg, S.G., Bezy, O., O'Neill, B.T., and Kahn, C.R. (2015). Role of PKCdelta in Insulin Sensitivity and Skeletal Muscle Metabolism. Diabetes.
3. Lee, K.Y., Russell, S.J., Ussar, S., Boucher, J., Vernochet, C., Mori, M.A., Smyth, G., Rourk, M., Cederquist, C., Rosen, E.D., et al. (2013). Lessons on conditional gene targeting in mouse adipose tissue. Diabetes *62*, 864-874.
4. Widrick, J.J., Maddalozzo, G.F., Lewis, D., Valentine, B.A., Garner, D.P., Stelzer, J.E., Shoepe, T.C., and Snow, C.M. (2003). Morphological and functional characteristics of skeletal muscle fibers from hormone-replaced and nonreplaced postmenopausal women. The journals of gerontology. Series A, Biological sciences and medical sciences *58*, 3-10.
5. Lee, K.Y., Singh, M.K., Ussar, S., Wetzel, P., Hirshman, M.F., Goodyear, L.J., Kispert, A., and Kahn, C.R. (2015). Tbx15 controls skeletal muscle fibre-type determination and muscle metabolism. Nat Commun *6*, 8054.
6. Winnay, J.N., Solheim, M.H., Dirice, E., Sakaguchi, M., Noh, H.L., Kang, H.J., Takahashi, H., Chudasama, K.K., Kim, J.K., Molven, A., et al. (2016). PI3-kinase mutation linked to insulin and growth factor resistance in vivo. J Clin Invest *126*, 1401-1412.
7. O'Neill, B.T., Lee, K.Y., Klaus, K., Softic, S., Krumpoch, M.T., Fentz, J., Stanford, K.I., Robinson, M.M., Cai, W., Kleinridders, A., et al. (2016). Insulin and IGF-1 receptors regulate FoxO-mediated signaling in muscle proteostasis. J Clin Invest *126*, 3433-3446.

8. Debosch, B.J., Chen, Z., Saben, J.L., Finck, B.N., and Moley, K.H. (2014). Glucose transporter 8 (GLUT8) mediates fructose-induced de novo lipogenesis and macrosteatosis. J Biol Chem *289*, 10989-10998.

9. Rockl, K.S., Hirshman, M.F., Brandauer, J., Fujii, N., Witters, L.A., and Goodyear, L.J. (2007). Skeletal muscle adaptation to exercise training: AMP-activated protein kinase mediates muscle fiber type shift. Diabetes *56*, 2062-2069.
