# Supplementary material for: Role of p110a subunit of PI3-kinase in skeletal muscle mitochondrial homeostasis and metabolism
Source: Nat Commun. 2019 Jul 30;10:3412. doi: 10.1038/s41467-019-11265-y (PMC6667496; doi:10.1038/s41467-019-11265-y)
Supplement: Supplementary file 3 — Reporting Summary [file 41467_2019_11265_MOESM3_ESM.pdf]

nature research

Corresponding author(s): C. Ronald Kahn

Last updated by author(s): Jun 18, 2019

Reporting Summary

Nature Research wishes to improve the reproducibility of the work that we publish. This form provides structure for consistency and transparency in reporting. For further information on Nature Research policies, see Authors & Referees and the Editorial Policy Checklist.

Statistics

For all statistical analyses, confirm that the following items are present in the figure legend, table legend, main text, or Methods section.

n/a	Confirmed	
☐	☒	The exact sample size (*n*) for each experimental group/condition, given as a discrete number and unit of measurement
☐	☒	A statement on whether measurements were taken from distinct samples or whether the same sample was measured repeatedly
☐	☒	The statistical test(s) used AND whether they are one- or two-sided<br>*Only common tests should be described solely by name; describe more complex techniques in the Methods section.*
☐	☒	A description of all covariates tested
☐	☒	A description of any assumptions or corrections, such as tests of normality and adjustment for multiple comparisons
☐	☒	A full description of the statistical parameters including central tendency (e.g. means) or other basic estimates (e.g. regression coefficient) AND variation (e.g. standard deviation) or associated estimates of uncertainty (e.g. confidence intervals)
☐	☒	For null hypothesis testing, the test statistic (e.g. *F*, *t*, *r*) with confidence intervals, effect sizes, degrees of freedom and *P* value noted<br>*Give P values as exact values whenever suitable.*
☒	☐	For Bayesian analysis, information on the choice of priors and Markov chain Monte Carlo settings
☒	☐	For hierarchical and complex designs, identification of the appropriate level for tests and full reporting of outcomes
☒	☐	Estimates of effect sizes (e.g. Cohen's *d*, Pearson's *r*), indicating how they were calculated

Our web collection on statistics for biologists contains articles on many of the points above.

Software and code

Policy information about availability of computer code

Data collection	N/A
Data analysis	All data were analyzed using GraphPad Prism 7 software and ImageJ was used to normalize the western blotting. Measurements were taken from distinct samples.

For manuscripts utilizing custom algorithms or software that are central to the research but not yet described in published literature, software must be made available to editors/reviewers. We strongly encourage code deposition in a community repository (e.g. GitHub). See the Nature Research guidelines for submitting code & software for further information.

Data

Policy information about availability of data

All manuscripts must include a data availability statement. This statement should provide the following information, where applicable:
- Accession codes, unique identifiers, or web links for publicly available datasets
- A list of figures that have associated raw data
- A description of any restrictions on data availability

The datasets generated during and/or analyzed during the current study are available from the corresponding author on reasonable request.

Field-specific reporting

Please select the one below that is the best fit for your research. If you are not sure, read the appropriate sections before making your selection.

☒ Life sciences ☐ Behavioural & social sciences ☐ Ecological, evolutionary & environmental sciences

Life sciences study design

All studies must disclose on these points even when the disclosure is negative.

Sample size	No sample-size calculations were performed. Number of sample used in this study was noted in the figure legend with a minimum of n = 3 .
Data exclusions	No data was excluded from the analyses.
Replication	All attempts at replication were successful.
Randomization	- All tissue samples were randomized.<br>- All animals were grouped randomly.
Blinding	In all experiments, except Western blot and qPCR, the investigators were blinded to the groups and samples manner.

Reporting for specific materials, systems and methods

We require information from authors about some types of materials, experimental systems and methods used in many studies. Here, indicate whether each material, system or method listed is relevant to your study. If you are not sure if a list item applies to your research, read the appropriate section before selecting a response.

Materials & experimental systems

n/a	Involved in the study	
☐	☒	Antibodies
☒	☐	Eukaryotic cell lines
☒	☐	Palaeontology
☐	☒	Animals and other organisms
☒	☐	Human research participants
☒	☐	Clinical data

Methods

n/a	Involved in the study	
☒	☐	ChIP-seq
☒	☐	Flow cytometry
☒	☐	MRI-based neuroimaging

Antibodies

Antibodies used	We provided information regarding production company, catalog number, dilution factor, etc., in the supplementary experimental procedures: Lysates were subjected to SDS-PAGE and blotted using following antibodies: p110α (CS4249, 1:1000), phospho-Akt (S473) (CS9271, 1:2000), phospho-ERK (CS9101, 1:2000), phospho-GSK3β (CS9336, 1:2000), phospho-4EBP1 (CS2855, 1:1000), phospho-ULK (S555) (CS5869, 1:1000), phospho-Drp1 (S637) (CS4867, 1:1000), phospho-FoxO1/3 (CS9464, 1:500), ERK (CS9102, 1:2000), GSK3β (CS9315, 1:2000), 4EBP1(CS9644, 1:500), LC3 (CS2775, 1:1000), Beclin- 1(CS3495, 1:1000), Drp1 (CS8570, 1:1000) and FoxO1 (CS2880, 1:500) from Cell Signaling, OPA1 (SC393296, 1:1000), GAPDH from Santa Cruz (SC25778, 1:5000), and citrate synthase (Ab96600, 1:1000), Mfn2 (Ab56889, 1:1000), and OXOPHOS (Ab110413, 1:1000) proteins from Abcam.
Validation	The use of antibodies were validated in the previous studies and published data.

Animals and other organisms

Policy information about studies involving animals; ARRIVE guidelines recommended for reporting animal research

Laboratory animals	Muscle-specific p110α or p110β knockout mice were generated by crossing mice carrying the Cre recombinase gene driven by the human alpha-skeletal actin (HSA) promoter (Jackson Laboratories Stock Number: 006149) with mice carrying either floxed p110α or p110β alleles in which exon 1 of p110α or exon 2 of p110β was flanked with loxP sites. Male mice at ages of 3-, 5-, 16- and 24- months of ages were used as noted in the figure legend.
Wild animals	N/A
Field-collected samples	N/A
Ethics oversight	All animal studies were performed using protocols approved by the Institutional Animal Care and Use Committee (IACUC) from Joslin Diabetes Center.

Note that full information on the approval of the study protocol must also be provided in the manuscript.
